# Supplementary material for: Exploring the Potential of siRNA Delivery in Acute Myeloid Leukemia for Therapeutic Silencing
Source: Nanomaterials (Basel). 2023 Dec 18;13(24):3167. doi: 10.3390/nano13243167 (PMC10745893; doi:10.3390/nano13243167)
Supplement: Supplementary file 1 [file nanomaterials-13-03167-s001.zip › nanomaterials-2687267-supplementary.pdf]

Supplementary Materials

Exploring the Potential of siRNA Delivery in Acute Myeloid Leukemia for Therapeutic Silencing

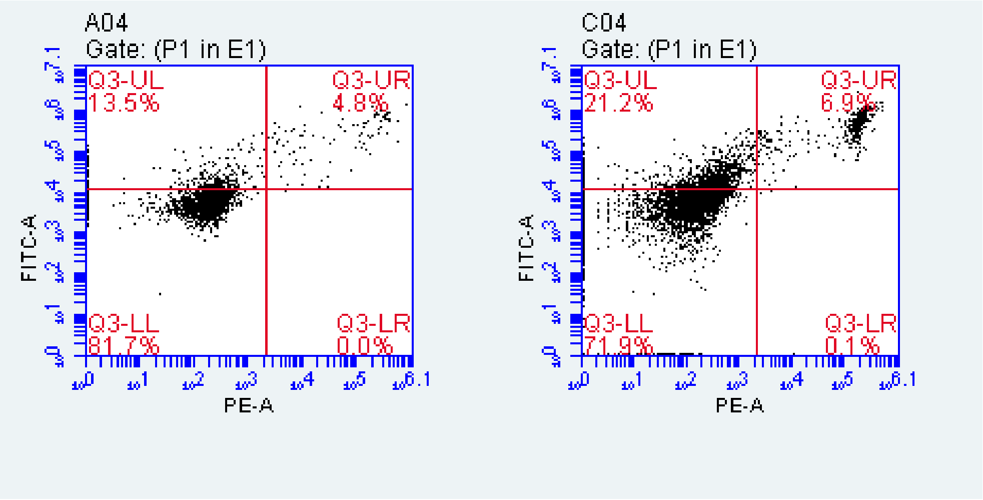

Figure S1: Flow cytometry histograms for non-treated (NT) cells and cells treated with surviving siRNA.
